# Clinical Notes

**Published:** 1898-06

**Authors:** Hugh Thompson

**Affiliations:** Shabbona, Illinois


### CLINICAL NOTES.

By HUGH THOMPSON, V. S.,
SHABBONA, ILLINOIS.

CASE I.—Gray draught mare, nine years old, weight 1400 pounds. Had foaled six hours before, and afterbirth had not followed. Removed the fœtal envelopes in about twenty minutes without difficulty, after which I gave her hypodermatically two grains of morphine, to prevent straining and to quiet any after-pains—a plan which I have followed for many years. The animal did well, and in about a week the owner put her into a cultivator and cultivated corn for two days. As she was turned out when not at work, and the day following her work in the cultivator was a rainy one, when she was taken up in the evening and put in the barn nothing specially was noted; she ate her evening ration of four quarts of oats and some hay, but the next morning ate very little, and refused her feed entirely the following two days. The owner, determining that something was wrong with her bladder, placed salt in the vaginal cavity, which was followed by violent straining and colicky pains. As the mare grew worse, I was summoned to see her. Found her bedewed with a cold sweat over the whole body, an anxious expression of countenance, and continually elevated the upper lip; the head, ears, legs, and hips cold; body on sides warm. Three blankets had been placed on her. Pulse almost imperceptible at the jaw; temperature normal; trembling of muscles of the shoulder, and forelimbs. Vulva, vaginal and rectal mucous membrane swollen and infiltrated; considerable straining, with three small passages of feces in five minutes.

*Treatment.* Gave stimulants of digitaline and nitroglycerine hypodermatically, and a little later, three grains of morphine. I injected the bowel with warm milk containing two drachms of carbolic acid, which was retained, and seemed to control straining. The mare lived just two hours after I reached her. Would like to know if the irritation and inflammation produced by the salt, producing shock, was the cause of death?

[The history indicates the presence of peritonitis, which was aggravated by the treatment applied by the owner.—EDITOR.]

CASE II.—Bay draught horse, six years old, weight 1500 pounds, suffering with severe flatulent colic, with excessive gaseous distention and intense pain. Fifteen grains of barium chloride were injected intravenously, which in a few minutes was followed by the passage of flatus, which continued irregularly for two hours, when the abdomen had become about normal in size. No feces were passed, and, fearing a return of the flatulence, gave a ball containing two drachms of aloin, one drachm of calomel, which produced purgation in eighteen hours, and continued for two days, when the owner, becoming alarmed, came to my office and reported the condition as well as a noticeable stiffness. I prescribed a mixture of tincture of opium, camphor, and turpentine, which controlled the purgation, and visited the horse the following day, as he was reported as breathing hard and not eating. Found the horse standing with his head pressed against the wall, badly bruised, forelimbs working continually; pulse at submaxillary imperceptible; artery felt like a piece of thread; vacant, staring expression; temperature, 103.5°; ears and legs cold, the former lopped; bowels had moved but once the preceding day. Unable to back him, and when efforts were made to raise the head, he pushed forward with all his strength. By placing a halter rope on each side of the stall, and the aid of several assistants, we were able to back him. After securing him he continued to press forward and continually moved the front limbs.

*Treatment.* Cannabis indica, digitalis, and salicylate of soda were successfully administered, which, in twenty minutes, was followed by quietude, hanging of the head, eye brighter; movements of the limbs ceased, and remained in this condition for two hours, when he fell. Gave him one-half grain each of strychnine and nitro-glycerine hypodermatically, which seemed to rouse him up somewhat, but death followed in a few hours. The writer would like to know if these were not unusual symptoms following superpurgation? This was my first case of the latter following the treatment as above described, which plan, where the barium chloride fails to bring any feces, I have followed with a ball of aloin and calomel.

[The condition described was such as would follow prolonged purgation, with the excessive irritation of the gastro-intestinal tract, and the subsequent disturbance of the nerve centres of the brain and spinal cord.—EDITOR.]

CASE III.—A case of atrophy of the shoulder muscles, where one-half grain of strychnine was injected hypodermatically in the postea spinatus muscle, and repeated at night, and the following morning three-fourths of a grain given, which was followed by acute symptoms of strychnine poisoning, violent spasmodic contraction of the muscles, throwing the horse from his feet to the ground. Intratracheal injections of one drachm of potassium iodide and three grains of sulphate of morphine caused relaxation of the muscles and gradual recovery from the symptoms. The subject was a grade Norman, four years old, and weighed about 1300 pounds. The writer has used strychnine in half-grain doses for several years in atrophied shoulders, and is at a loss to know why the increased quarter of a grain should have produced the symptoms described.

[Strychnine is a remedy that should always be used with the greatest caution, particularly in ascending doses. The great difference in the susceptibility of animals is one factor; idiosyncrasies of certain patients; the age and quality of the drug vary much, and its solubility in tablet-form is variable.—EDITOR.]

